# Tocilizumab-associated posterior reversible encephalopathy syndrome in giant-cell arteritis – case report

**DOI:** 10.1186/s12883-021-02231-7

**Published:** 2021-06-22

**Authors:** Michaela Butryn, Sabine Mewes, Eugen Feist, Oliver Beuing, Christian Müller, Jens Neumann

**Affiliations:** 1 Institute of Cognitive Neurology and Dementia Research, Otto von Guericke University Magdeburg, Magdeburg, Germany; 2 German Centre for Neurodegenerative Diseases, Magdeburg, Germany; 3 Department of Neurology, Otto von Guericke University Magdeburg, Leipziger Straße 44, 39120 Magdeburg, Germany; 4 Department of Rheumatology, |Helios clinic, Gommern, Germany; 5 Department of Radiology, Otto von Guericke University Magdeburg, Magdeburg, Germany; 6 Department of Gastroenterology, Hepatology und Infectiology, Otto von Guericke University Magdeburg, Magdeburg, Germany

**Keywords:** Tocilizumab, Posterior reversible encephalopathy syndrome, Giant cell arteritis

## Abstract

**Backround:**

We describe one of the first cases of a Posterior reversible encephalopathy syndrome (PRES) under tocilizumab as treatment of Giant cell arteritis (GCA).

**Case presentation:**

A 65-year-old female with known GCA and treatment with Tocilizumab (TCZ) developed a convulsive epileptic seizure for the first time. MRI was suggestive of PRES and an associated left sided occipital hemorrhage. Extensive high blood pressure values were not detected. The patient recovered within a week and no further seizures occurred under anticonvulsive medication.

**Conclusion:**

PRES during the treatment with Tocilizumab hasn’t been described in GCA so far. There are single reports of an association between TCZ and PRES in other entities. Thus, a link between interleukin-6 and the integrity of the vasculature could be considered. The clinical consequence should be a stringent blood pressure monitoring in the ambulant setting of patients receiving TCZ.

## Backround

Giant cell arteritis (GCA) is the most common vasculitis in adult Caucasians affecting large and medium sized vessels leading to critical ischemia. In approximately 50% of the patients polymyalgia rheumatica is coexistent. Headache including claudicatio masticatoria are the most frequent symptoms. Vessels of all body regions can be affected in principle, however, ophthalmic manifestations are most feared and frequent, while involvement of extra- and intracranial arteries with subsequent strokes are rare complications [1].

Tocilizumab (TCZ) is a humanized monoclonal antibody against the interleukin-6 receptor (IL-6R), which was first approved for the treatment of rheumatoid arthritis (RA) [2]. The approval for GCA followed in 2017, further indications are systemic-onset juvenile idiopathic arthritis, cytokine release syndrome and others [3]. The most relevant adverse drug reactions are infections, hepatotoxicity, neutropenia and gastrointestinal perforations [4].

Posterior reversible encephalopathy syndrome (PRES) is a rare, mainly hypertension-associated condition with symptoms comprising headache, seizures, confusion, disturbed vision and consciousness as correlate of oedema, in particular affecting the occipital lobes. An association of PRES with some autoimmune disorders and especially with immunosuppressive treatments (mostly cyclosporine and tacrolimus) is known [5].

Here we present one of the first cases of PRES under tocilizumab *for the treatment of. a GCA.*

## Case presentation

We describe a 65-year-old Caucasian female with known GCA since 2018 with (ultrasound detected) affection of the left axillary artery, left temporal artery and right sided carotid artery. Under ongoing therapy with low-dose prednisolone (4 mg per day) and tocilizumab (162 mg per week subcutaneously since the initial diagnosis), she was admitted to our department of neurology after occurrence of a convulsive epileptic seizure in October 2019. The patient showed only mild symptoms with a left sided latent sensorimotor hemiparesis and slight cognitive impairment. A mild hypertension was known in the patient**’**s history without any specific medication. During the transport and the admission to our hospital the patient developed moderate hypertensive values up to a maximum of 170/90 mmHg.

**Fig. 1:**
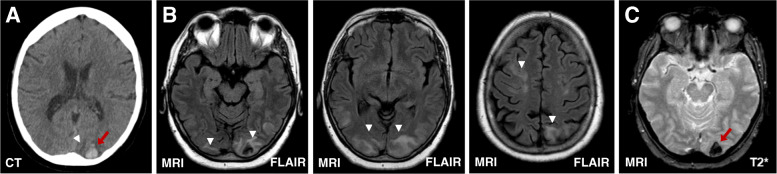
Vasogenic edema and occipital haemorrhage in PRES. **a** The initial CT scan detected a left located occipital haemorrhage (red arrow) as well as occipital accentuated subcortical hypodensities (white arrow head) indicative of an edema. **b** MRI scan confirmed the CT hypodensities as edema (white arrow heads) and the haemorrhage in the T2*-weighted gradient echo (**c**) (red arrow)

Computer tomography (CT) of the brain revealed bilateral distinct occipital hypodense lesions with left sided hemorrhagic features. Brain magnetic resonance imaging (MRI) showed bilateral extent T2 hyperintense lesions occipital, parietal, frontal and cerebellar without diffusion restriction with the known hemorrhagic changes on the left side (Fig. 1). We performed CT-angiography which was inconspicuous. At that date there was no evidence of active inflammation (halo sign) in the vertebral and temporal arteries using ultra sound. The electroencephalogram (EEG) showed bilateral occipital slowing without epileptic activity. The analysis of the cerebrospinal fluid (CSF) was unremarkable, especially without any evidence of JC-virus.

After immediate initiation of antihypertensive and anticonvulsive (Levetiracetam 2 **×** 750 mg) medication the patient recovered from neurological symptoms within a week and no further seizures appeared since then.

Diagnosis of PRES was established by typical clinical findings and the specific changes in the brain MRI. Several cases of PRES have been reported under other immunosuppressive regiments. So we consider that TCZ - as part of a multi-hit hypothesis in combination with slightly increased blood pressure values against the backdrop of the autoimmune disease itself - caused PRES [6]. Thus, we discontinued TCZ application and continued oral glucocorticosteroids with remaining remission of GCA.

## Discussion and conclusion

Tocilizumab-associated neurological complications have been reported previously. In 2009 a patient with RA developed a leukoencephalopathy and more recently a patient with juvenile idiopathic arthritis exhibited PRES in 2018 [7, 8]. Another case described the occurrence of PRES after infusion of anti-BCMA directed CAR T cells and application of tocilizumab but also of high dose methylprednisolone and hydrocortisone to limit the cytokine release syndrome. The authors discussed the cytokine release storm itself as responsible for PRES but did not consider the immunosuppressive therapies as possible reason [9]. Especially hemorrhage in PRES, like in our case seems to be associated to immunosuppression [10]. A presentation of PRES in GCA-patients treated with TCZ has not been reported to date.

The association between TCZ and PRES has not been investigated yet, neither in humans nor in an appropriate animal model, speculating a link between cerebral vasculature and the influence of cytokines, e.g. IL-6. It is suggested that elevated IL-6 levels in brain microvascular endothelial cells lead to a loss of integrity, which was inhibited by IL-6 receptor blockade [11, 12]. But that is counterintuitive referring to the presented case.

A link between IL-6 and the activation of endothelial cells is more conceivable. IL-6 can induce endothelin-1 (ET-1), which serves as the most potent mediator for vasoconstriction. IL-6 binds soluble sIL-6R and builds a complex with gp130 which in turn could elevate endothelin-1 expression in endothelial cells [13]. TCZ binds IL-6R and inhibits IL-6 mediated actions and thus could lead to decreased endothelin-1 concentrations. Impaired (exhausted) autoregulation of cerebral arteries is thought to be a part of the pathophysiology behind PRES, thus it is tempting to speculate that lower ET-1 concentration impairs the function of endothelial smooth muscle cells. Brain vessels of the posterior circulation are more susceptible to develop edema under high blood pressure, due to less adrenergic innervation [14]. They might be even more sensitive to a lower ET-1 concentration, potentially leading to compromised regulation of higher blood pressure values. In order to get beyond speculation and due to rare cases in humans, a suitable animal model to investigate the association between blocking IL-6 receptors and PRES should be considered. In order to get beyond speculation and due to rare cases in humans, a suitable animal model to investigate the association between blocking IL-6 receptors and PRES should be considered.

## Data Availability

Not applicable.

## Abbreviations

BCMA: B-cell maturation antigen
CAR: Chimeric antigen receptor
CSF: Cerebrospinal fluid
CT: Computer tomography
EEG: Electroencephalogram
ET-1: Endothelin-1
GCA: Giant cell arteritis
gp130: Glycoprotein 130
IL-6: Interleukin-6
IL-6R: Interleukin-6 receptor
JC-virus: John Cunningham-virus
mg: Milligram
MRI: Magnetic resonance imaging
PRES: Posterior reversible encephalopathy syndrome
RA: Rheumatoid arthritis
TCZ: Tocilizumab
